# Antares I: a Modular Photobioreactor Suitable for Photosynthesis and Bioenergetics Research

**DOI:** 10.1007/s12010-023-04629-0

**Published:** 2023-07-24

**Authors:** Mónica Rodríguez-Bolaños, Gloria Vargas-Romero, Girian Jaguer-García, Zhaida I. Aguilar-Gonzalez, Verónica Lagos-Romero, Héctor V. Miranda-Astudillo

**Affiliations:** Departamento de Biología Molecular y Biotecnología, Instituto de Investigaciones Biomédicas, Universidad Nacional Autónoma de México, Mexico City, Mexico

**Keywords:** Photobioreactor, Microalgae production, Spectrum optimization

## Abstract

Oxygenic photosynthesis is responsible for most of the fixation of atmospheric CO_2_. The microalgal community can transport atmospheric carbon into biological cycles in which no additional CO_2_ is created. This represents a resource to confront the actual climate change crisis. These organisms have evolved to adapt to several environments and different spectral distribution of light that may strongly influence their metabolism. Therefore, there is a need for development of photobioreactors specialized in addressing spectral optimization. Here, a multi-scale modular photobioreactor made from standard glass materials, ad hoc light circuits, and easily accessible, small commercial devices is described. The system is suitable to manage the principal culture variables of research in bioenergetics and photosynthesis. Its performance was tested by growing four evolutionary-distant microalgal species with different endosymbiotic scenarios: *Chlamydomonas* *reinhardtii* (*Archaeplastida*, green primary plastid), *Polytomella parva* (*Archaeplastida*, colorless plastid), *Euglena gracilis* (*Discoba*, green secondary plastid), and *Phaeodactylum tricornutum* (*Stramenophiles*, red secondary plastid). Our results show an improvement of biomass production, as compared to the traditional flask system. The modulation of the incident light spectra allowed us to observe a far-red adaptation in *Euglena* *gracilis* with a difference on paramylon production, and it also significantly increased the maximal cell density of the diatom species under green light. Together, these confirm that for photobioreactors with artificial light, manipulation of the light spectrum is a critical parameter for controlling the optimal performance, depending on the downstream goals.

**Supplementary Information:**

The online version contains supplementary material available at 10.1007/s12010-023-04629-0.

## Introduction

Climate change is an ongoing process that will change the relations between all living organisms on Earth. Normally, more than 90% of planetary carbon is stored in algae, vegetation, and coral reefs as biomass or organic compounds; besides, the actual accumulation of gaseous CO_2_ in the atmosphere produced by human activity intensifies the greenhouse effect, which has increased the average temperature of the planet over the last few decades [1]. As primary producers and capable of performing photosynthesis, microalgae absorb sunlight (photons) and convert inorganic carbon (from the atmosphere or industrial emissions) into organic carbon biomass. Thus, an exponential advance in research for alternative energies, e.g., biofuels (in contrast to fossil fuels), and efforts to increase atmospheric CO_2_ fixation have been achieved over the last decades [2]. However, nature developed this strategy a long time ago during the so-called Great Oxidation Event, which occurred about 2.4 billion years ago, when ancestors of cyanobacteria dramatically changed the environment massively fixing atmospheric CO_2_ and using sunlight energy to produce organic matter and O_2_ [3]. More complex photosynthetic organisms arose later by different endosymbiotic events. The first was the engulfment of a cyanobacterium by a heterotrophic eukaryote. From that, the three contemporary algal lineages emerged: chlorophytes, glaucophytes, and rhodophytes. Red algae (rhodophytes) appeared around 1.2 billion years ago, and green algae (chlorophytes) circa 0.75 billion years ago [4]. Molecular evidence suggests that glaucophytes were the earliest to branch off from the common ancestor [5]. Members of the chlorophytes and rhodophytes were then engulfed by independent eukaryotic hosts resulting in lineages with secondary plastids [6]. These events gave rise to an extensive variety of relations between newly born organelles and the hosts. Consequently, these organisms evolved to adapt to several environments and light qualities. The sequestration of CO_2_ in microalgal systems has some advantages like better efficiency in light utilization, higher growth rates, and larger biomass production, as compared to terrestrial plants [7], e.g., to produce 1 kg of microalgal biomass, 1.83 of kg carbon is used [8]. These values illustrate that microalgae represent an interesting alternative to antagonize the ongoing crisis in climate change [9].

During the last decades, research to increase production of biomass has led to construct large open cultivation facilities and indoor photo bioreactors (PBR) [10–12]. With the advent of novel molecular engineering tools to improve the capacity of CO_2_ fixation [13] or the production of high-value metabolites, e.g., vitamins, pigments, and fatty acids [14–17], several cultivation conditions need to be tested. The main variables that need to be overseen during microalgae cultivation are temperature, light intensity, air/CO_2_ intake, and light spectrum quality control. Most of the available commercial PBRs control one, or more, of these variables. Nevertheless, multi-scale bioreactors for screening purposes and comparative studies are lacking, especially those that allow light spectrum control. The aim of this work was to develop a scalable, low-cost PBR and validate its suitability to control the main physical parameters, focusing on performing subsequent bioenergetic and photosynthesis research.

## System Design

**Fig. 1:**
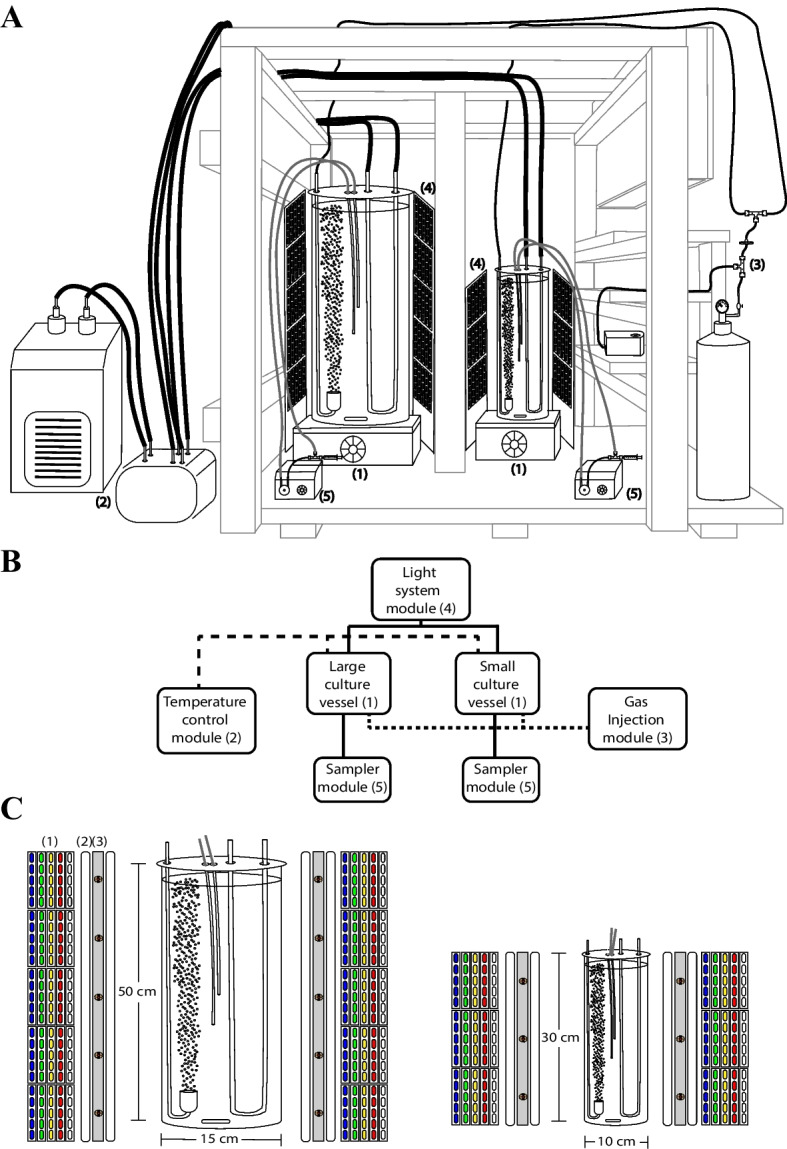
Schematic representation of the modular photobioreactor Antares I. **A** Diagram of the complete system in the scaffold. For clarity the system is divided into five modules: (1) culture vessels, (2) temperature control, (3) gas injection, (4) light system, and (5) sampler module. **B** General instrumentation scheme of the modular photobioreactor; the connectivity of the different modules is shown. Numbers indicate the same modules shown in panel **A**. **C** Scheme of the light system distribution surrounding each culture vessel. (1) 0.5W 5630-type LEDs. (2) FLCLED 7 W/45 cm. (3) 3-W LED far-red (720–730 nm). Please refer to point 2 in text for details

Figure 1 shows a schematic illustration of the modular PBR. Several small devices are combined into the scaffold of the modular system to feed and maintain the central growth glass vessels (1.7 and 7.5 L) (Fig. 1). The featured system regulates temperature, light intensity, CO_2_/air injection, and light spectrum. For convenience, the full system will be divided into minor modules for description: temperature, gas injection, light system, and sampler modules. Agitation inside each vessel is achieved using a combination of magnetic stirrer/gas-column flow which guarantees the homogeneity of the culture. Details from each component of the system are described in supplemental Table 1.

### Temperature Regulation Module

A 24-L water tank connected to a C-250 water chiller (*Boyu*) regulates the temperature (16–35 °C) of the whole system. A SP-2500 water pump (*Boyu*) recirculates the liquid (1400 L/h) from the tank to the refrigeration system and back (Fig. 1, panel A(2)). Two extra SP-1800 water pumps (*Boyu*) inject water (700 L/h) trough individual glass heat interchanger U-tubes inside each culture vessel (Fig. 1, panel C).

### Gas Injection Module

The two culture vessels can optionally be fed with air and/or CO_2_. Two A-807 air pumps (*Elite-Hagen*) or an in situ CO_2_ generator (*ZRDR*) injects the corresponding gas to the vessels. The injected gases are sterilized using a 0.22-µm PVFD filter (Corning) connected to each pipeline (Fig. 1, panel A(3)). A GA-105 ceramic/glass CO_2_ Atomizer (*Dymax*) disperses the gas flowing into the media (Fig. 1, panel C). The gas injection is measured with a bubble counter (*ZRDR*) and the volume/time flow (µL/s) is calculated according to [18].

### Light System Module

The ad hoc light system consists of the following: a primary system formed by four 4000-K white LED lamps per vessel (FLCLED 4W/25 cm) for the small vessel and four 4000-K white LED lamps (FLCLED 7W/45 cm) for the larger one. Spectral modification is accomplished by two larger groups of 12-V 0.5-W 5630-type color LEDs, blue (455–465 nm), green (515–525 nm), and red (635–645 nm). Six modules (5 LEDs/module) per color surround the small vessel and ten modules the larger one (Fig. 1, panel C). Additionally, sixteen 3-W LEDs far-red (720–730 nm) surround the culture vessels, 10 LEDs for the large and 6 for the small vessel, respectively. Optionally, 2700–3000-K and 5500–6500-K LED modules are used to maintain a basal illumination for the rest of the spectrum (Fig. 1, panel A(4) and panel C). An electrical diagram of the ad hoc light system is shown in Figure S1. Emission spectra from all the light sources were acquired with a Plano-Convex lens Collimator coupled to a SMA-E Spectrometer (*Thunder Optics*, France), using the Spectragryph acquisition software (https://www.effemm2.de/spectragryph/index.html).

### Sampler Module

A peristaltic pump (*Intlab*) recirculates the culture through a sterile heat-resistant silicon tube (3-mm internal diameter). A sterile syringe, connected with a 3-way Luer lock, is used to collect 1 mL of sample each time (Fig. 1, panel A(5)).

## Material and Methods

### Algal Strain Cultures and Membrane Preparation

*Chlamydomonas reinhardtii* cell-wall–less mutants *cw15 mt* + were grown in continuous light conditions, illuminated with a 4000-K white LED lamp at an intensity of 50 µmol photons m^−2^ s^−1^ (400–700 nm). The liquid mineral Tris-minimum-phosphate medium (TMP) pH 7.0 [19] was supplemented with a mix of vitamins (biotin 10^–7^%, B12 vitamin 10^–7^%, and B1 vitamin 2 × 10^–5^% (w/v)). The culture was fed with a continuous air injection of 20 µL/s. Cells were collected at the middle of the logarithmic phase by a 10-min centrifugation step at 7000 × *g* and stored at − 70 °C until use. All the following steps were performed at 4 °C. The resulting cell pellet was resuspended in MET buffer (280 mM mannitol, 100 mM EDTA, 10 mM Tris-HCl pH 7, and 0.1% BSA) and then disrupted by sonication two times (30 s each, setting 5 W, 50% output) using a Branson-450 sonifier, as described in [20]. Total membranes were obtained by differential centrifugation, 3000 × *g*/10 min to remove unbroken cells, followed by a 20,000 × *g*/10 min centrifugation step to recover total membranes. The sample was stored at − 70 °C until use.

The colorless alga *Polytomella* sp. (198.80 Culture Collection of Algae, University of Göttingen, Germany, identical to *Polytomella parva*) was grown in liquid mineral Tris-acetate-phosphate medium (TAP 30 mM acetate) pH 7.0 [21] supplemented with a mix of vitamins (biotin 10^–7^%, B12 vitamin 10^–7^%, and B1 vitamin 2 × 10^–5^% (w/v)). The culture was fed with a continuous air injection of 50 µL/s. No mechanical agitation was used. Cells were collected at the middle of the logarithmic phase by a 10-min centrifugation step at 7000 × *g* and stored at − 70 °C until use. All the following steps were performed at 4 °C. The cellular pellet was resuspended in SPT Buffer (0.3 M sucrose, 4 mM potassium EDTA, and 20 mM Tris, pH 7.2), and cells were broken mechanically with a Potter homogenizer with five to six gentle manual strokes of the Teflon pestle as described in [22]. Total membranes were obtained by differential centrifugation, 1000 × *g*/10 min to remove unbroken cells, followed by a 17,000 × *g*/10 min centrifugation step to recover total membranes. Samples were stored at − 70 °C until use.

*Euglena gracilis* (SAG 1224-5/25) was obtained from the University of Göttingen (Sammlung von Algenkulturen, Germany). Cells were grown in continuous light conditions, illuminated with a 4000-K white LED lamp at an intensity of 50 µmol photons m^−2^ s^−1^ (400–700 nm). TMP medium supplemented with a vitamin mix was fed with a continuous CO_2_ injection of 5.6 µL/s. Cells were collected at the middle of the logarithmic phase by a 10-min centrifugation step at 7000 × *g* and stored at –70 °C until use. All the following steps were performed at 4 °C. Cells were washed with SHE buffer (sucrose 250 mM, HEPES 10 mM, EDTA 1 mM, pH 7.3) and disrupted with glass beads (500-µm diameter) using five 15-s vortex cycles with a 1-min rest. The sample was centrifuged (2000 × *g*/10 min) to remove unbroken cells and total membranes were collected by centrifugation (8500 × *g*/10 min) and stored at –70 °C until use.

*Phaeodactylum tricornutum* Pt1 8.6 (CCMP 2561) cells were grown in continuous light conditions, illuminated with a 4000-K white LED lamp at an intensity of 100 µmol photons m^−2^ s^−1^ (400–700 nm). Liquid ESAW medium [23] containing NaNO_3_ (46.8 mg L^−1^) and NaH_2_PO_4_·H_2_O (3.1 mg L^−1^) was supplemented with the same mix of vitamins described above. The culture was fed with continuous air injection (40 µL/s). The cells were collected at the middle of the logarithmic phase by a 30-min centrifugation step at 7000 × *g* and stored at –70 °C until use. All following steps were performed at 4 °C. Cells were resuspended in SoHE buffer (0.66 M Sorbitol, 6 mM EDTA, 50 mM HEPES, 5 mM MnCl_2_, 1 mM MgCl_2_, pH 7.3) in a 1:5 cells:buffer ratio (vol/vol). The suspension was mechanically broken with glass beads (500-µm diameter) by vortexing three times for 5 min, with 5-min resting on ice between each period. The sample was centrifuged (2500 × *g*/10 min) to remove unbroken cells and total membranes were collected by centrifugation (14,000 × *g*/10 min) and stored at –70 °C until use.

### Growth Curves

Culture development was followed by two independent methods: chlorophyll *a* absorption was measured at 675 nm [24] on a VE-51000 UV Spectrometer (VELAB), and cell count was performed in a Neubauer double ruled Counting Chamber. Cell images were acquired with a Canon EOS 700D camera coupled to a CH2 modular biological microscope (Olympus). Dissolved O_2_ was measured with a D09100 Oxygen Electrode (RCYAGO). Volumetric oxygen mass transfer coefficient (*kLa*) was determined as previously described in [25].

### Native and Denaturing Electrophoresis

Total membranes were solubilized with *n*-dodecyl-β-d-maltoside (β-DDM) 2% in solubilization buffer (SB) containing 50 mM Tris–HCl, 1.5 mM MgSO_4_, 100 mM NaCl, and 10% glycerol, pH 8.4. All steps were performed at 4 °C. The mixture was incubated with gentle agitation for 2 h, and centrifuged at 30,000 × *g* for 30 min. After discarding the insoluble material, supernatants were subjected to BN-PAGE [26]. Native-PAGE was carried out in 4–12% acrylamide gradient gels. In gel activity for complex I was carried out as described by [27]. Protein concentration was determined by the Bradford method (Biorad).

### Spectrometry Analysis

Spectra at room temperature were obtained directly from the collected cultured cells. Absorbance spectra were acquired on a Cary 60 UV-Vis Spectrophotometer (Agilent), while fluorescence spectra (excitation* λ* = 470 nm) were obtained using a USB2000 + Ocean Optics Spectrometer coupled to a LS-450 Series Blue LED Pulsed Light Source (Ocean Optics Inc., Dunedin, FL, USA).

### Paramylon Quantification

Total paramylon production was determined as described in [28] with slight modifications. Briefly, 8 × 10^6^ cells from the stationary phase of the culture were collected by centrifugation at 2200 × *g*/20 min, and the cellular pellet was resuspended by vortex in 1.0 mL of SDS 1% and incubated 15 min in boiling water; after this period, the sample was incubated 10 min in ice and the paramylon pellet was collected by centrifugation at 2200 × *g*/20 min. This procedure was repeated one time, and an additional SDS 1% wash was performed. The clean paramylon pellet was resuspended by vortex in 1.0 mL of NaOH 1.0 M, and a sample of 50 µL was transferred to a new tube where 600 µL of phenol 5% solution was added followed by 2.5 mL of concentrated H_2_SO_4_. The sample was incubated 25 min at 25 °C and the absorbance at 490 nm was determined. The concentration was estimated with a calibration curve (0–100 µg) of dextrose (USP standard).

## Results

### Antares I Allows Growth of Several Photosynthetic and Non-photosynthetic Eukaryotes

**Fig. 2:**
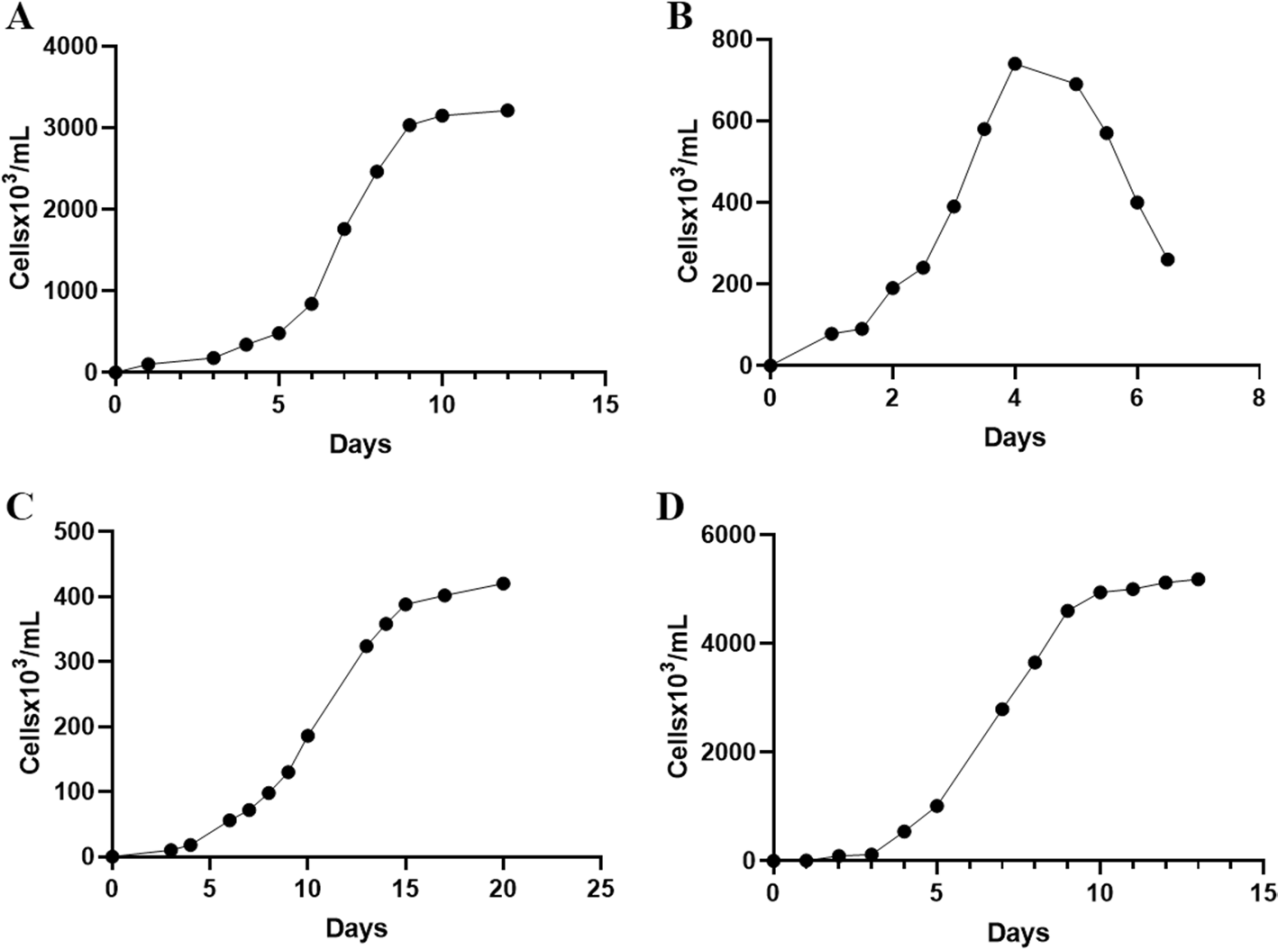
Growth curves of 4 microalgal species in the modular photobioreactor Antares I. *Chlamydomonas reinhardtii* in TMP medium plus air injection of 20 µL/s with 50 µmol photons m^−2^ s^−1^ (400–700 nm) (**A**), *Polytomella parva* in TAP medium plus air injection of 50 µL/s (**B**), *Euglena gracilis* in TMP medium plus 5.6 µL/s CO_2_ injection with 50 µmol photons m^−2^ s^−1^ (400–700 nm) (**C**), and *Phaeodactylum tricornutum* in ESAW medium plus air injection of 40 µL/s with 100 µmol photons m^−2^ s.^−1^ (400–700 nm) (**D**)

**Fig. 3:**
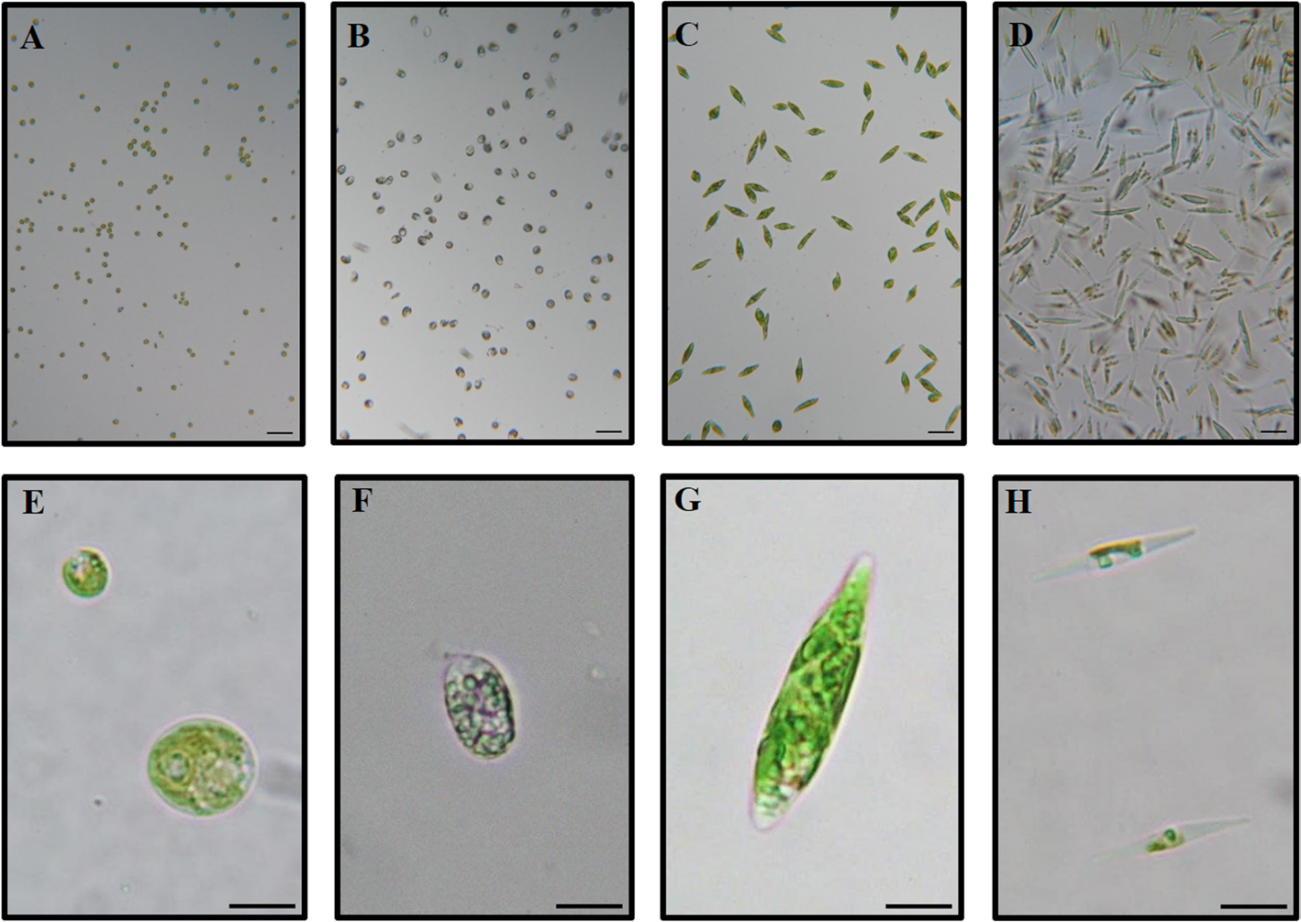
Cellular morphology of grown microalgal species. Field view (upper panels) and zoomed view of cells (lower panels) in the middle of logarithmic phase, *Chlamydomonas reinhardtii* (**A**, **E**), *Polytomella parva* (**B**, **F**), *Euglena gracilis* (**C**, **G**), and *Phaeodactylum tricornutum* (**D**, **H**). The scale bar is 10 µm

A diagram of the designed system is shown in Fig. 1; its modular architecture allows us to grow different culture volumes, from 0.5 to 7.5 L with light modulations from 10 to 300 µmol photons m^−2^ s^−1^ (400–700 nm) on white light and 10 to 100 µmol photons m^−2^ s^−1^ on a specific color: blue (455–465 nm), green (515–525 nm), red (635–645 nm), and far-red (720–730 nm) (Fig. 1, panel C). The principal culture parameters determined for all the species are presented in Table 1. The maximal cell density varies among the cultivated species, being approximately 3.2 × 10^6^, 0.78 × 10^6^, 0.4 × 10^6^, and 5.2 × 10^6^ cells mL^-1^ for *C. reinhardtii*, *P. parva*, *E. gracilis*, and *P. tricornutum*, respectively (Fig. 2). An example of the increase of cellular density against time of a culture of *E. gracilis* is shown in Supplemental Fig. 2. The cells from the different organisms showed physical properties characteristic for each species (Fig. 3), e.g., mobility was observed for all species, except the diatom. To follow the culture development of photosynthetic species, the same samples used for cell counting were used to follow chlorophyll *a* absorption (675 nm). Similar growth curves to those described in Fig. 2 were obtained (Fig. S3, panels A–C). A nonlinear correlation of both growth curves was obtained for each species, indicating a proportional relation between the number of cells and the absorption of chlorophyll *a* (Fig. S3, panels D–I).

**Table 1:** Culture parameters from several microalga species growth in the designed PBR and flask system under similar culture conditions

	Determines in the presented system			Literature values			References
Organism (condition)	Doubling time (days)	Growth rate (days^−1^)	Maximum cell density (cells/mL)	Doubling time (days)	Growth rate (days^−1^)	Maximum cell density (cells/mL)	
*Chlamydomonas reinhardtii* (white 50 µmol photons m^−2^ s^−1^, air 20 µL/s)	1.30	0.43	3.2 × 10^6^	0.58 150 µmol photons m^−2^ s^−1^ 2.1–2.2 60 µmol photons m^−2^ s^−1^, 0.005% CO_2_	—	3.1 × 10^7^ CO_2_ enriched air, 200 µmol photons m^−2^ s^−1^	[29–31]
*Polytomella parva* (TAP medium, air 50 µL/s)	0.96	0.68	0.78 × 10^6^	—	—	0.18 × 10^6^ TAP medium	[32]
*Polytomella parva* (TAP medium, air 37 µL/s)	1.15	0.60	0.41 × 10^6^	—	—	—	—
*Polytomella parva* (TAP medium, air 25 µL/s)	2.41	0.51	0.28 × 10^6^	—	—	—	—
*Euglena gracilis* (white 50 µmol photons m^−2^ s^−1^ CO_2_ 5.6 µL/s)	2.23	0.31	0.4 × 10^6^	—	0.30 NI µmol photons m^−2^ s^−1^ 1.09 16 h/8 h (light/dark) intensity of 145 µmol photons m^−2^ s^−1^	0.18 × 10^6^ 12 h/12 h (light/dark) 1-month culture 50 µmol photons m^−2^ s^−1^	[33–35]
*Euglena gracilis* (far-red 50 µmol photons m^−2^ s^−1^ CO_2_ 5.6 µL/s)	2.76	0.25	0.4 × 10^6^	—	—	—	—
*Euglena gracilis* (white 50 µmol photons m^−2^ s^−1^ CO_2_ 10.2 µL/s)	2.07	0.33	0.82 × 10^6^	—	—	—	—
*Euglena gracilis* (far-red 50 µmol photons m^−2^ s^−1^ CO_2_ 10.2 µL/s)	1.99	0.32	0.79 × 10^6^	—	—	—	—
*Phaeodactylum tricornutum* (white 50 µmol photons m^−2^ s^−1^ air 40 µL/s)	1.8	0.17	5.2 × 10^6^	0.75 1–2% CO^2^ 20-W fluorescent lamp 1.00 40 µmol photons m^−2^ s^−1^ 16 °C pH 8.5	0.5–0.7 1–2% CO^2^ 20-W fluorescent lamp 0.99 40 µmol photons m^−2^ s^−1^ 16 °C pH 8.5	5.0 × 10^6^ 40 µmol photons m^−2^ s^−1^ 16 °C pH 8.5	[36, 37]
*Phaeodactylum tricornutum* (green 50 µmol photons m^−2^ s^−1^ air 40 µL/s)	1.13	0.62	14.9 × 10^6^	—	—	—	—

*NI*, not indicated

To evaluate the efficiency of the system on the distribution of the injected gases, the volumetric oxygen mass transfer coefficient (*kLa*) was determined. A value of 21.89 h^−1^ at an air flux of 50 µL/s was obtained (Fig. S4). The effect of this gas distribution was evaluated by growing *P.* *parva* under fully respiratory conditions (e.g., acetate as carbon source with different air fluxes) and *E.* *gracilis* grown phototrophically using different CO_2_ fluxes. A direct effect of the air flux over the maximal cellular density was observed on *Polytomella* cultures (Fig. S5A and Table 1), interestingly, similar oxygen consumption curves were obtained under the three conditions (Fig. S5B). In a similar way, a larger amount of CO_2_ flux increased the maximal cellular density up to 0.82 × 10^6^ cells mL^-1^ of phototrophic *Euglena* cultures; although a decrease of the time to reach the stationary phase was also observed (Fig. S5C and Table 1), the oxygen concentration among all the *Euglena* growth curves remained between 92 and 99% (Fig. S5D) reflecting the oxygen production by the photosynthetic machinery. In summary, the PBR allows us to grow microorganisms with different physical and biological properties and to estimate the cell density measuring chlorophyll *a* absorption.

### The Cultured Organisms Show Integrity of Their Bioenergetic Machinery

**Fig. 4:**
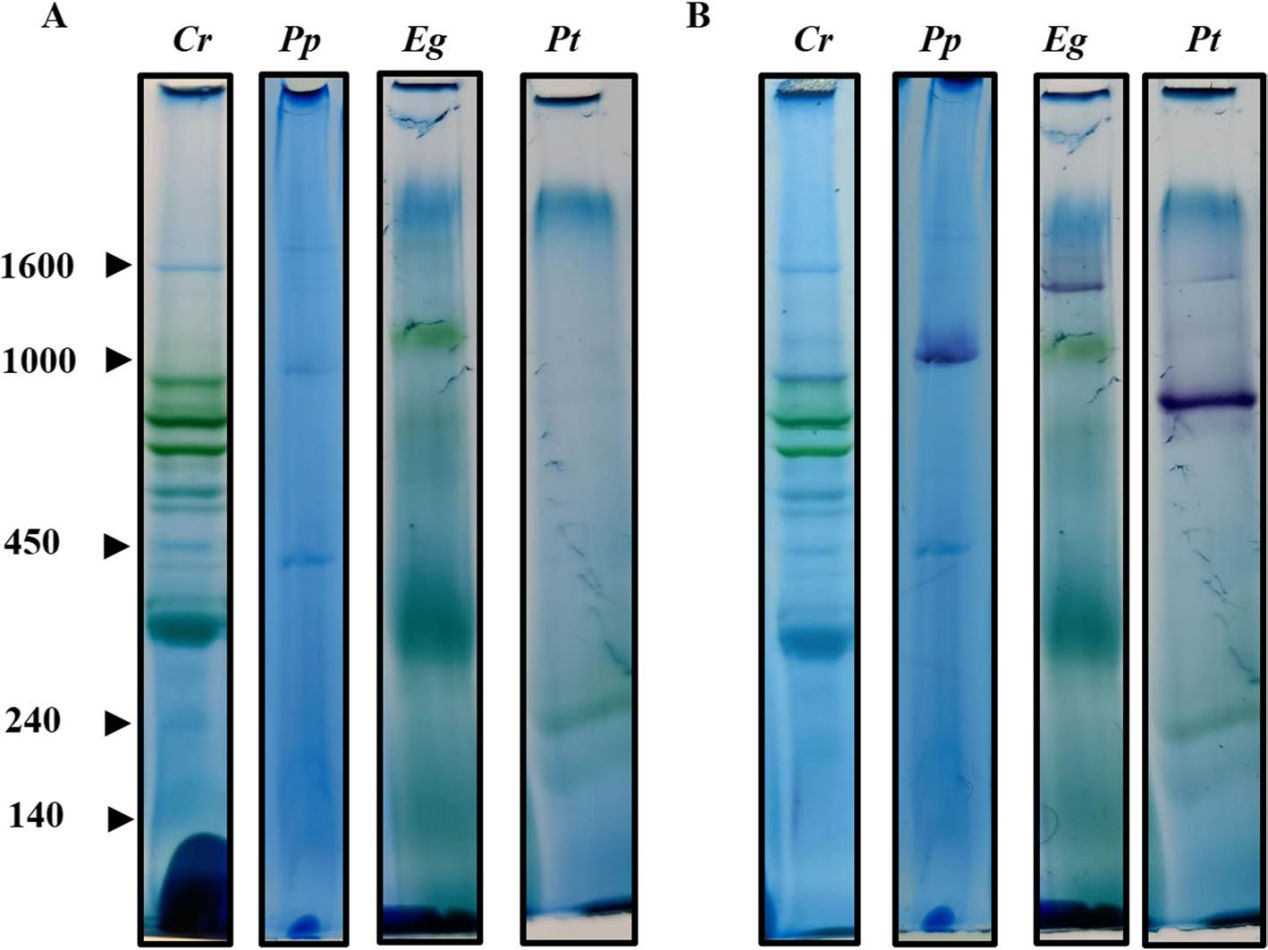
Bioenergetic complexes from the studied microalgal species. Total membranes (0.4 µg) were solubilized with *n*-dodecyl-β-D-maltoside (DDM) (2%) and resolved by BN-PAGE in a 4–12% polyacrylamide gradient gel (**A**). In-gel NADH-dehydrogenase activity; the BN-gel was incubated in the presence of NADH and nitro blue tetrazolium chloride (NBT) to evidence the presence of mitochondrial complex I (**B**). *Cr*, *Pp*, *Eg*, and *Pt* correspond to *Chlamydomonas reinhardtii, Polytomella parva*, *Euglena gracilis*, and *Phaeodactylum tricornutum*, respectively. Molecular masses in kDa are marked on the left side

To evaluate the integrity of the bioenergetic machinery of each microalga, total membranes were prepared. The energy-transducing complexes, i.e., OXPHOS and photosynthetic complexes, were extracted from membranes with mild detergents, e.g., *n*-dodecyl-β-D-maltoside (β-DDM), and then subjected to BN-PAGE (Fig. 4). The presence of pigment-proteins complexes was directly observed in all species, except for *P. parva* (Fig. 4A). A difference in size between the photosynthetic complexes was evident, and probably related to different photosystem types or to the stability and structural differences between species. The presence of mitochondrial complex I was revealed with in-gel NADH:NBT dehydrogenase activity (Fig. 4B). The size of mitochondrial complex I also varied among the different species, as has been previously described [38–40]. The presence of both photosynthetic and mitochondrial complexes suggests that the integrity of the bioenergetic machineries is not compromised in the culture conditions used.

### The Featured System Allows the Evaluation of Incoming Light Spectra on Cellular Development

**Fig. 5:**
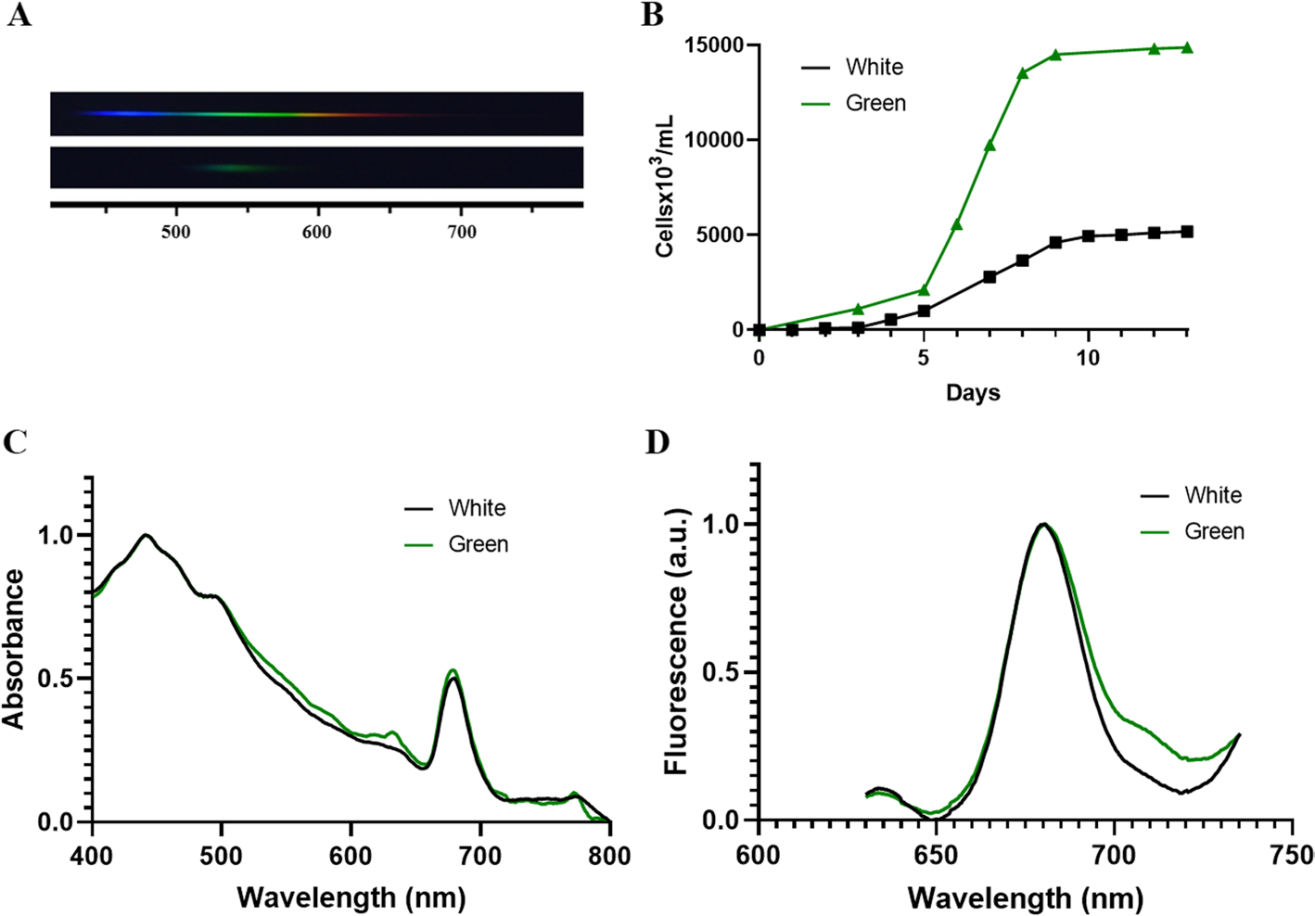
Effect of different light spectra on *Phaeodactylum tricornutum* growth. **A** Graphic frame of the spectra used for both culture conditions, white light (upper panel) and green light (lower panel). **B** Growth curves of *P. tricornutum* under different light sources: black line, white light; green line, green light (515–525 nm). **C** Room temperature absorption spectra of *P. tricornutum* cell growth under different light sources: black line, white light; green line, green light (515–525 nm). **D** Room temperature fluorescence spectra (excitation *λ* = 470 nm) of *P. tricornutum* cell growth under different light sources: black line, white light; green line, green light (515–525 nm)

**Fig. 6:**
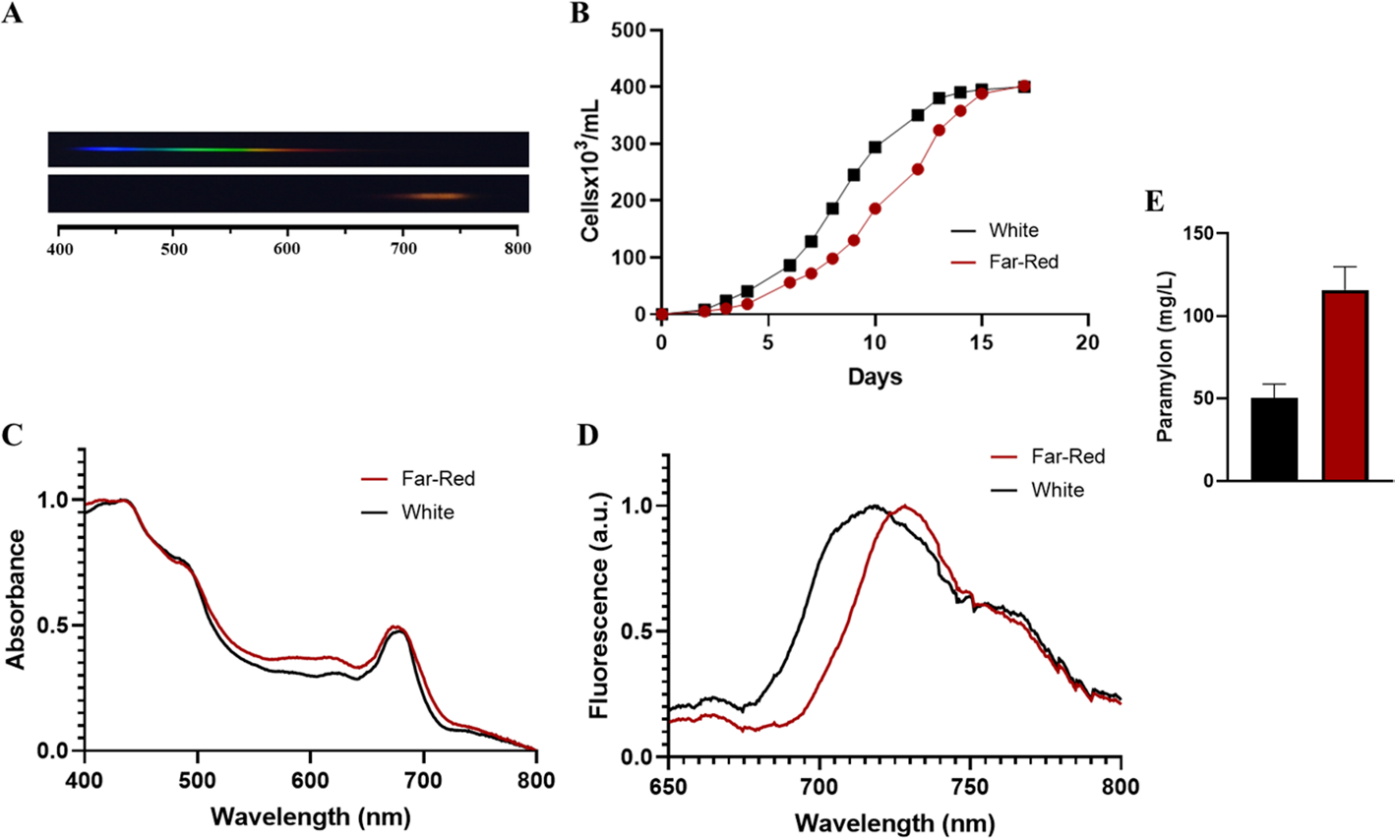
Effect of different light spectra on *Euglena gracilis* growth. **A** Graphic frame of the spectra used for both culture conditions, white light (upper panel) and far-red light (lower panel). **B** Growth curves of *E. gracilis* under different light sources: black line, white light; red line, far-red light (720–730 nm). **C** Room temperature absorption spectra of *E. gracilis* cell growth under different light sources: black line, white light; red line, far-red light (720–730 nm). **D** Room temperature fluorescence spectra (excitation *λ* = 470 nm) of *E. gracilis* cell growth under different light sources: black line, white light; red line, far-red light (720–730 nm). **E** Paramylon (β-(1,3)-glucan) determination from *E. gracilis* growth under different light regimes: black column, white light; red column, far-red light (720–730 nm).

To explore how our system allows to follow the effect of different light spectra on cellular development, *P. tricornutum* and *E. gracilis* were grown maintaining the same total photon concentration (100 and 50 µmol photons m^−2^ s^−1^, respectively), but using green light (515–525 nm, Fig. 5A) and far-red light (720–730 nm, Fig. 6A), respectively. A remarkable difference in the growth curves was observed for *P. tricornutum*, green light increased the maximal cellular level as compared to white light (Fig. 5B). To search for changes in pigment properties, absorption and fluorescence spectra were obtained. Slight changes were observed in absorption spectra, where a small increase in the 520–600- and 700–710-nm regions is detectable, and an additional 630-nm peak is present in green light–grown cells (Fig. 5C). Therefore, a small increase in red emission (> 700 nm) is observed for the green light culture; nevertheless, the same signal maxima are present for both conditions (Fig. 5D). *E. gracilis* was capable to grow using a far-red light as the primary source. Similar growth curves were determined as compared to white light (Fig. 6B). An absorption increase in the 550–650-nm region can be observed (Fig. 6C). Interestingly, using a different spectra of incident light, a remarkable ~ 10-nm shift in the fluorescence maximal emission was observed when comparing both cultures (Fig. 6D). To search for changes in storage polysaccharide production, total paramylon (β-1,3-glucan) content was determined for steady-state cells grown using both lights. Cells adapted to far-red light presented larger paramylon rate production compared with white light cultures (Fig. 6E).

Taken together, our results indicate that the described system is useful for the culture of different photosynthetic and non-photosynthetic eukaryotes under optimal conditions. It also allows the manipulation of incident light spectra. As shown in Figs. 5 and 6, this is an important parameter that modulates cell development that induces several metabolic changes, setting the point for further bioenergetics and photosynthesis research in these organisms.

## Discussion

Culture conditions must be controlled to assure the reproducibility of a given metabolic state or metabolite production in microalgal biotechnology. The determined microalga growth curves have similar shapes as those of other organisms such as bacteria [41] and yeast [42]; however, the time to reach the stationary state varies widely among the studied species, from 4 days in *Polytomella* to up to 15–20 days in *Euglena* (Fig. 2). This fact emphasizes the importance to have a quick way to estimate cell density in the growing cultures, especially in the absence of high-cost cell counters. Chlorophyll *a* is a widely distributed pigment present in the majority of photosynthetic organisms and its absorption is usually proportional to cell number in many phytoplankton species [24]. Here, we determined a correlation between chlorophyll *a* absorption and cell density for each of the studied photosynthetic species (Fig. S3); thus, a quick absorbance determination at 675 nm allows us to estimate cell numbers with confidence for each species. The determined doubling time for *Chlamydomonas* culture (1.3 days) is half of the previously reported for flask culture using similar media and light conditions [29]. On the other hand, *Polytomella* reached a maximum cell density of 7.8 × 10^5^ cells/mL, which is larger than the 1.8 × 10^5^ cells/mL value previously reported for wide-bottom flask culture [32] (Table 1); to our knowledge, this is the first report of a *Polytomella* culture in a non-flask related system.

The volumetric oxygen mass transfer coefficient (*kLa*) is an important parameter in the PBRs that reflects an adequate gas distribution to optimize the conditions for proliferation of cells. The determined *kLa* value is comparable with those of other systems suitable for microalgal culture [43, 44] reflecting a good gas distribution. In fully aerobic *Polytomella* cultures, the oxygen injection, but not its distribution, seems to be the limiting factor; this is observed in the similar shape of the oxygen consumption among the three evaluated conditions (Fig. S5A, lower panel), and evidence the importance of the *kLa* as a crucial step in the design, operation, and scaleup of bioreactors [45]. In the case of phototrophically growth *Euglena*, the increase of CO_2_ derived into an larger cell density of the culture which is in line with the large capacity of CO_2_ fixation in different microalga species [46].

Cell bioenergetic machineries are the main energy producers in microalgae: OXPHOS in mitochondria and photosynthesis in chloroplasts. Although the two processes can be studied apart, in vivo they both cooperate and self-regulate, e.g., OXPHOS provides the ATP for carbon fixation when the photosynthetic machinery is compromised in *Chlamydomonas* [47, 48]. Cooperation between mitochondria and chloroplasts has also been observed in *Euglena* [49] and *Phaeodactylum* [50] species. The fact that our cultivated organisms exhibited typical morphologies (Fig. 3) and the presence of photosynthetic and mitochondrial complexes (Fig. 4) indicates that, under the standardized conditions, our system is useful to routinely grow photosynthetic and non-photosynthetic eukaryotes.

Light spectra vary naturally with time and localization during each day [51]. In some places, depending on the latitude, this variation is even larger during the progress of the year, and can be further affected by atmospheric factors [52]. Therefore, to assure survival, photosynthetic organisms have improved light harvesting to adapt to a large variety of niches. Beyond assuring life, this adaptation to light is also reflected in metabolic changes. For instance, exposure to spectra of variable composition affects the organoleptic characteristics of chili pepper (*Capsicum annuum* L.) [53]. Also, blue light showed effects on the production of carotenoids and other pigments in crops [54]. As in other better studied photosynthetic organisms, microalgal growth is affected by differences in light source like fluorescent lamps, RGB LEDs, or white LEDs [55, 56]. Additionally, irradiance and wavelength also affects production of metabolites like fatty acids, pigments, and carotenoids [57–59]. Consequences on growth can be observed in the *Phaeodactylum* species, where the maximal density is notably increased when this diatom was grown under green light (515–525 nm) (Fig. 5B). Previous reports proposed that silicon enhances growth under green light [60] but all our test were performed in silicon-free artificial medium. The spectroscopic analysis indicated only a small absorption increase in the 520–600-nm region and an extra peak at 630 nm (Fig. 5C), not accompanied by changes in the maximal fluorescence signal (Fig. 5D). These slight differences in absorption capacity may be related to a pigment content modification, especially because the chlorophyll *c*/*a* ratio increased from 0.54 to 0.68 between white and green light cultures. This is in agreement with previous reports, where no major spectroscopic differences were detected when this species was grown under different light wavelengths [61]. Globally, this indicates that the remarkable change in growth speed is related to a pigment content modifications and the ability of green photons to penetrate deeper into high-density cultures compared to other wavelengths [62] rather than to drastic structural changes in the photosynthetic machinery. While green light is usually less effectively utilized for growth because of the so-called green gap between 500 and 600 nm, the marine chlorophyte *Picochlorum* sp. is able to grow efficiently under green light reaching comparable biomass yields to red and white light controls [63].

The longest wavelength captured by antenna chlorophylls that can efficiently drive the reaction centers is ca. 680–700 nm, known as the “red limit” of oxygenic photosynthesis [64]. Interestingly, *Euglena gracilis* was able to grow under far-red light (720–730 nm) reaching a similar cellular density as under white light (Fig. 6B). Our spectroscopic analysis revealed no considerable differences between the absorption properties of both type of cells, e.g., white and far-red grown cells (Fig. 6C); nevertheless, a remarkable ~ 10-nm red shift in the fluorescence maximum signal appeared (Fig. 6D). A similar spectroscopic red shift has been observed in the cyanobacterium *Chroococcidiopsis thermalis* grown under far-red light that has been related to a change in the chlorophyll *a*/*f* ratio in the far-red absorbing antenna [65] that increases both absorption and quantum efficiency of Photosystem II [66]. A recent model proposes an adaptation of chlorophyll *a* in eukaryotic reaction centers, rather than an exchange of pigments; along this line, the reaction center is preorganized for charge separation in the far-red region by specific chlorophyll–pheophytin pairs [67]. Nevertheless, the specific far-red light adaptive mechanism of the *Euglena* photosynthetic machinery remains obscure, and for sure leaves the door open for further research in the peculiarities of its light-induced adaptation. The metabolic effect of light spectra was also observed in the production of paramylon, the storage carbohydrate in this species, where the far-red light induced a larger paramylon production (Fig. 6E), a similar, but less remarkable effect on this carbohydrate production that has been observed in *Euglena* grown under red light [68]; nevertheless, to our knowledge, this is the first report of the effect of larger wavelength (720–730 nm) on β-1,3-glucan production in *E. gracilis*.

### Concluding Remarks

The discovery of new microalgal species and the development of new molecular techniques to modify them may lead to new strategies to confront the actual climate crisis. For this purpose, several culture conditions need to be tested in a fast and reliable way. Our featured system fulfils this task and is especially designed to control and manipulate the main variables of microalgal culture, particularly allowing a flexible modulation of light incident spectra, and aims to implement downstream bioenergetic and photosynthesis research. The total cost of the system is about 3000 US dollars (Table S1); nevertheless, we have to note that the larger cost is related to the temperature module, where the water chiller represents more than one-third of the total cost of the system. The present work may represent a guide to build a photobioreactor with the versatility to exchange the components depending on the availability and price of the small commercial devices. Unfortunately, most commercially available systems leave the spectral modulation outside their scope, but our affordable system gives the opportunity to expand the tools available for bioenergetic and photosynthesis research to a larger scientific community.

### Supplementary Information

ESM 1 **Figure S1.** Electrical diagram of the *ad-hoc* light system of the modular photobioreactor Antares I. Three different electrical power sources distribute the energy to the different light modules. (1) Four 4000K FLCLED 4W/45cm are directly connected to the 110V 15A line. (2) Ten 3W LEDs far-red (720-730 nm) are connected to a 700A, 11-36V, 25.3W direct current source. (3) Eighty different 0.5W 5630-type color LEDs modules (blue (455-465 nm), green (515-525 nm) and red (635-645 nm), 2700-3000K (yellow) and 5500-6500K (white)) rise from the 30A, 12V, 360W direct current source. (4) Six 3W LEDs far-red (720-730 nm) are connected to a 700A, 9-24V, 16.8W direct current source. (5) Four 4000K FLCLED 4W/45cm are directly connected to the 110V 15A line. (PNG 498 kb)

High resolution image (TIF 14451 kb)

ESM 2 **Figure S2.** Cellular density increase for the culture of *E. gracilis*. The photograph series illustrate the culture development of *E. gracilis* in TMP medium plus 5.6 µL/s CO_2_ injection with 50 µmol photons m^-2^ sec^-1^ (400–700 nm). Numbers indicate days of the culture. (PNG 2558 kb)

High resolution image (TIF 11779 kb)

ESM 3 **Figure S3.** Growth curves for 3 microalgal species in the modular photobioreactor Antares I followed by their absorption at 675 nm. *Upper panels*: *Chlamydomonas reinhardtii* in TMP medium plus air injection of 20 µL/s with 50 µmol photons m^-2^ sec^-1^ (400–700 nm) (A), *Euglena gracilis *in TMP medium plus 5.6 µL/s CO_2_ injection with 50 µmol photons m^-2^ sec^-1^ (400–700 nm) (B) and *Phaeodactylum tricornutum* in ESAW medium plus air injection of 40 µL/s with 50 µmol photons m^-2^ sec^-1^ (400–700 nm) (C). *Middle panels*: nonlinear correlation between the data of cell count growth (see Figure 2) and 675nm absorption growth curve. *Chlamydomonas reinhardtii* (D), *Euglena gracilis* (E) and *Phaeodactylum tricornutum* (F). *Lower panels*: equations of the determined third-degree order polynomial function for each species. *Chlamydomonas reinhardtii* (G), *Euglena gracilis* (H) and *Phaeodactylum tricornutum* (I). (PNG 449 kb)

High resolution image (TIF 16835 kb)

ESM 4 **Figure S4.** Volumetric oxygen mass transfer coefficient (*kLa*) for the PBR. Dissolved oxygen quantification after the start of air injection inside oxygen-lacking media (A). Linear regression of relative oxygen concentration [(C*-C)/C*] against time. C*: Saturation oxygen concentration (8.2 mg/L);* C*: measured oxygen concentration by time. (PNG 187 kb)

High resolution image (TIF 9377 kb)

ESM 5 **Figure S5. **Growth curves for 2 microalgal species in the modular photobioreactor Antares I with different gas injections. *Polytomella parva* in TAP medium with increasing air fluxes 25 (▲), 37 (■) and 50 (●) µL/s) (A). Dissolved oxygen concentration of the growth curves from panel A, air fluxes 25 (Δ), 37 (□) and 50 (○) µL/s) (B). *Euglena gracilis *in TMP medium plus 5.6 µL/s (open symbols) and 10.2 µL/s (closed symbols) of CO_2_ injection under different light sources, 50 µmol photons m^-2^ sec^-1^, black lines: white light, red lines: far-red light (720-730 nm) (C). Dissolved oxygen concentration of the growth curves from panel C (D).(PNG 297 kb)

High resolution image (TIF 15064 kb)

ESM 6 (DOCX 14 kb)

## Data Availability

Not applicable
