# Supplementary figures and images for: Antares I: a Modular Photobioreactor Suitable for Photosynthesis and Bioenergetics Research
Source: Appl Biochem Biotechnol. 2023 Jul 24;196(4):2176–95. doi: 10.1007/s12010-023-04629-0 (PMC11035454; doi:10.1007/s12010-023-04629-0)

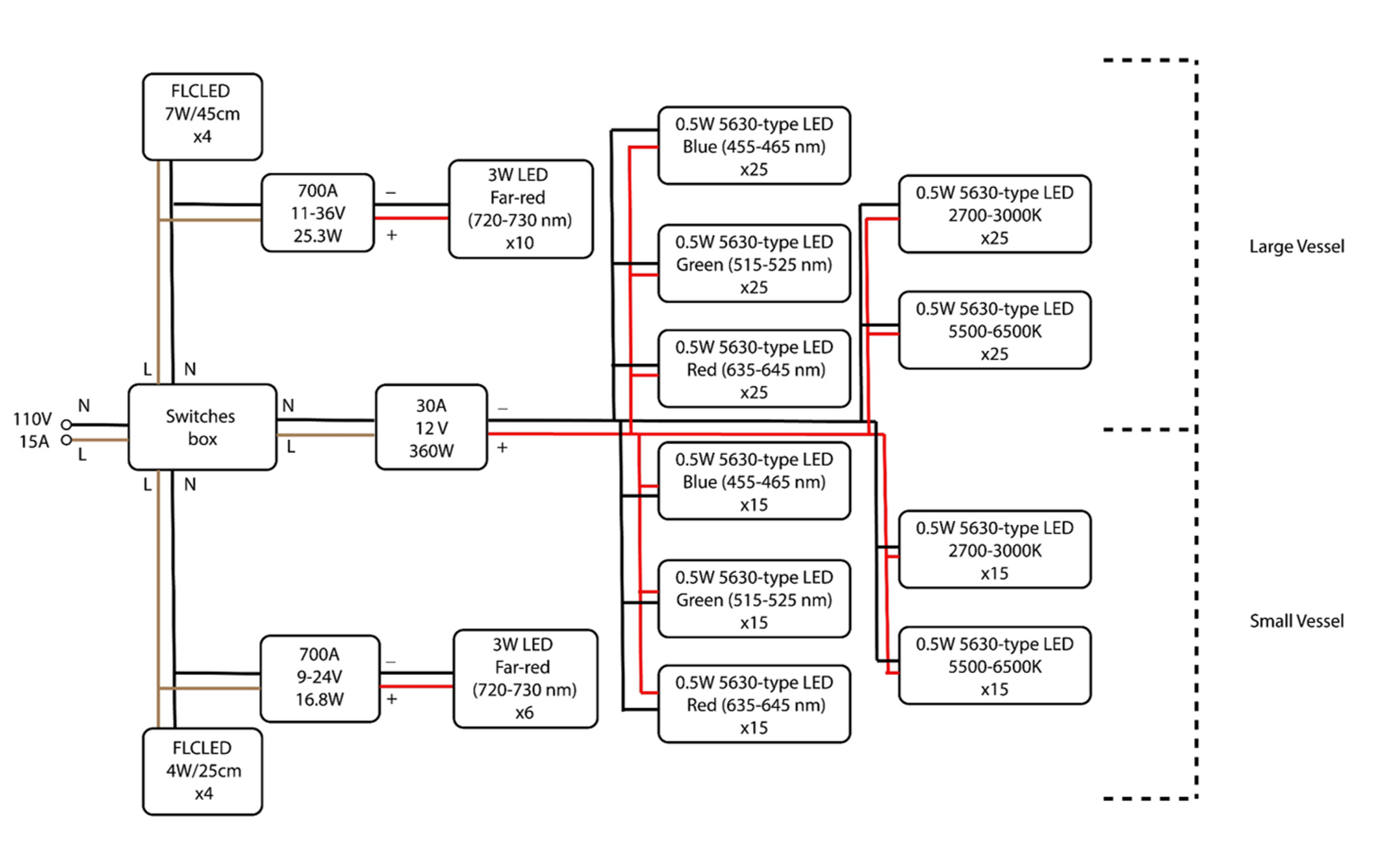

Supplement: Supplementary file 1 — Figure S1. Electrical diagram of the ad-hoc light system of the modular photobioreactor Antares I. Three different electrical power sources distribute the energy to the different light modules. (1) Four 4000K FLCLED 4W/45cm are directly connected to the 110V 15A line. (2) Ten 3W LEDs far-red (720-730 nm) are connected to a 700A, 11-36V, 25.3W direct current source. (3) Eighty different 0.5W 5630-type color LEDs modules (blue (455-465 nm), green (515-525 nm) and red (635-645 nm), 2700-3000K (yellow) and 5500-6500K (white)) rise from the 30A, 12V, 360W direct current source. (4) Six 3W LEDs far-red (720-730 nm) are connected to a 700A, 9-24V, 16.8W direct current source. (5) Four 4000K FLCLED 4W/45cm are directly connected to the 110V 15A line. (PNG 498 kb) [file 12010_2023_4629_Fig7_ESM.png]

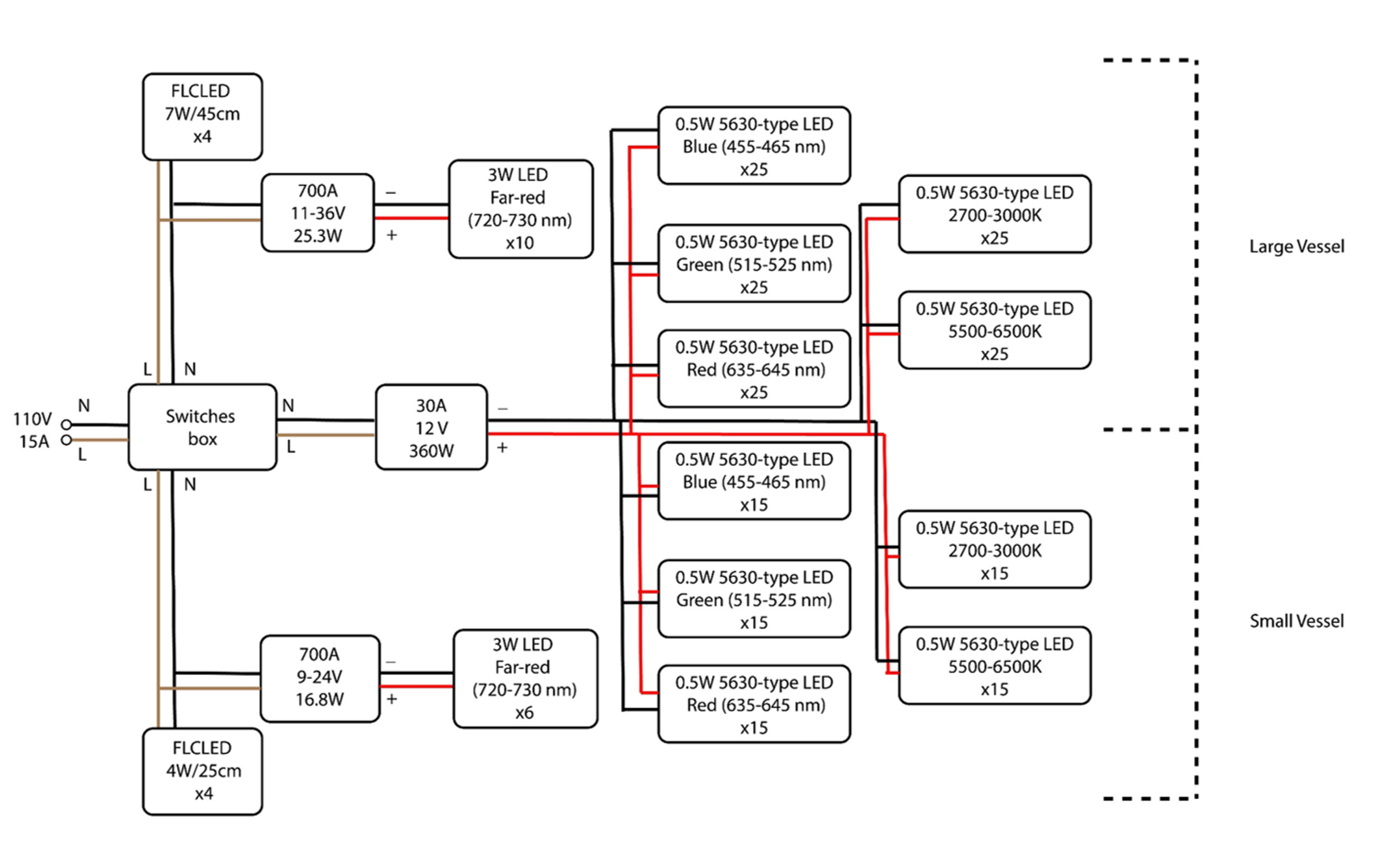

Supplement: Supplementary file 2 — High resolution image (TIF 14451 kb) [file 12010_2023_4629_MOESM1_ESM.tif]

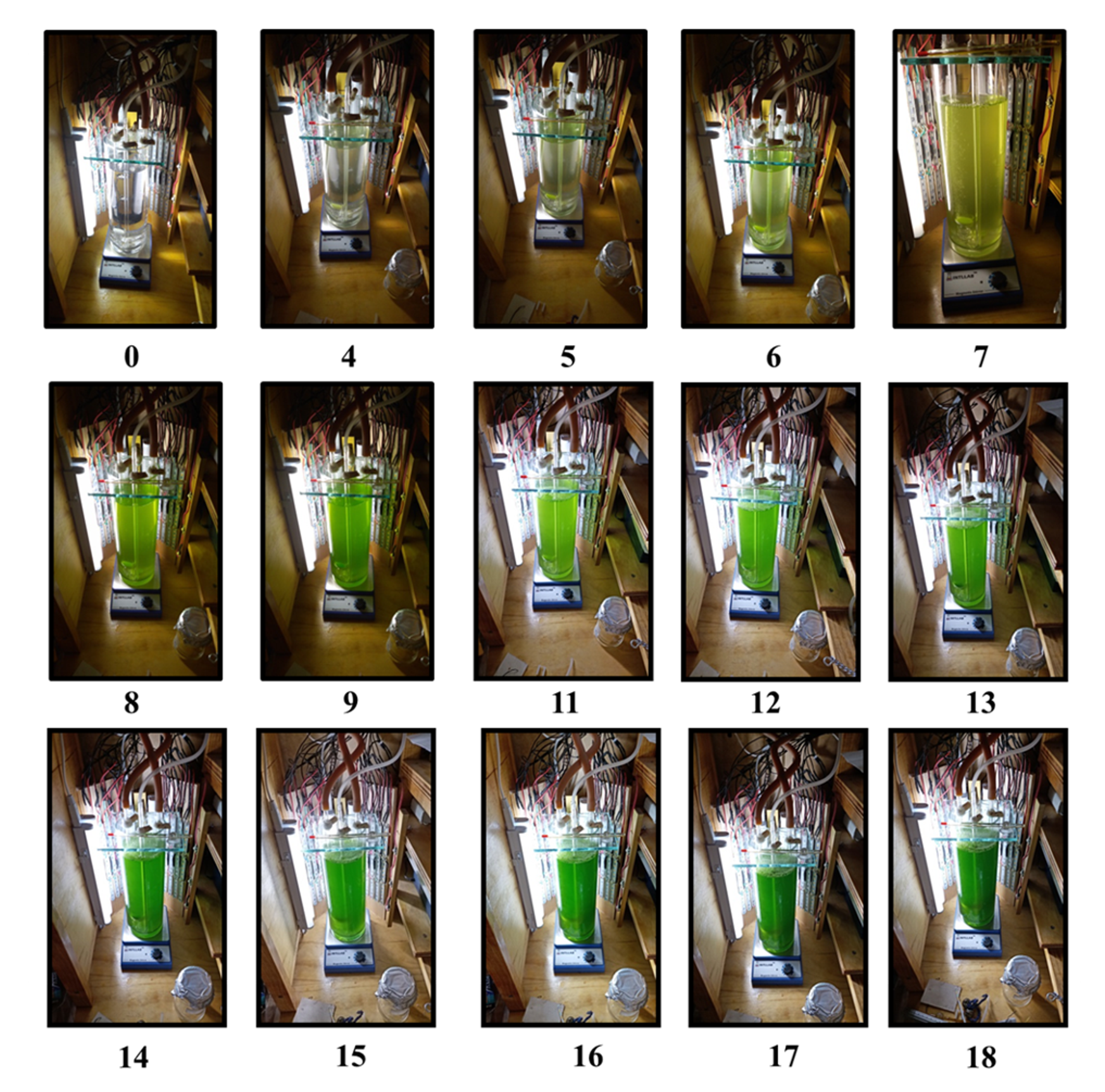

Supplement: Supplementary file 3 — Figure S2. Cellular density increase for the culture of E. gracilis. The photograph series illustrate the culture development of E. gracilis in TMP medium plus 5.6 µL/s CO2 injection with 50 µmol photons m-2 sec-1 (400–700 nm). Numbers indicate days of the culture. (PNG 2558 kb) [file 12010_2023_4629_Fig8_ESM.png]

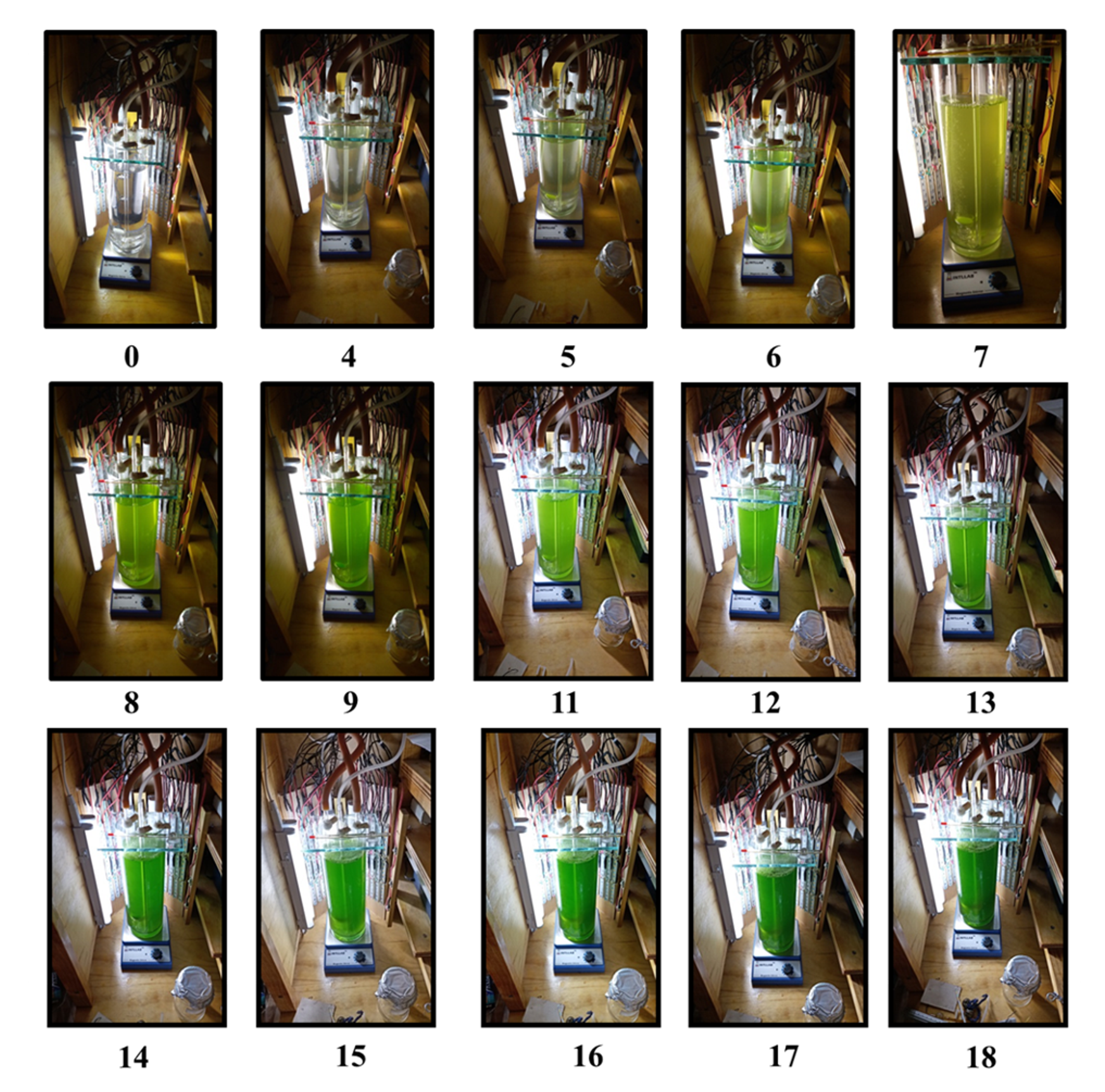

Supplement: Supplementary file 4 — High resolution image (TIF 11779 kb) [file 12010_2023_4629_MOESM2_ESM.tif]

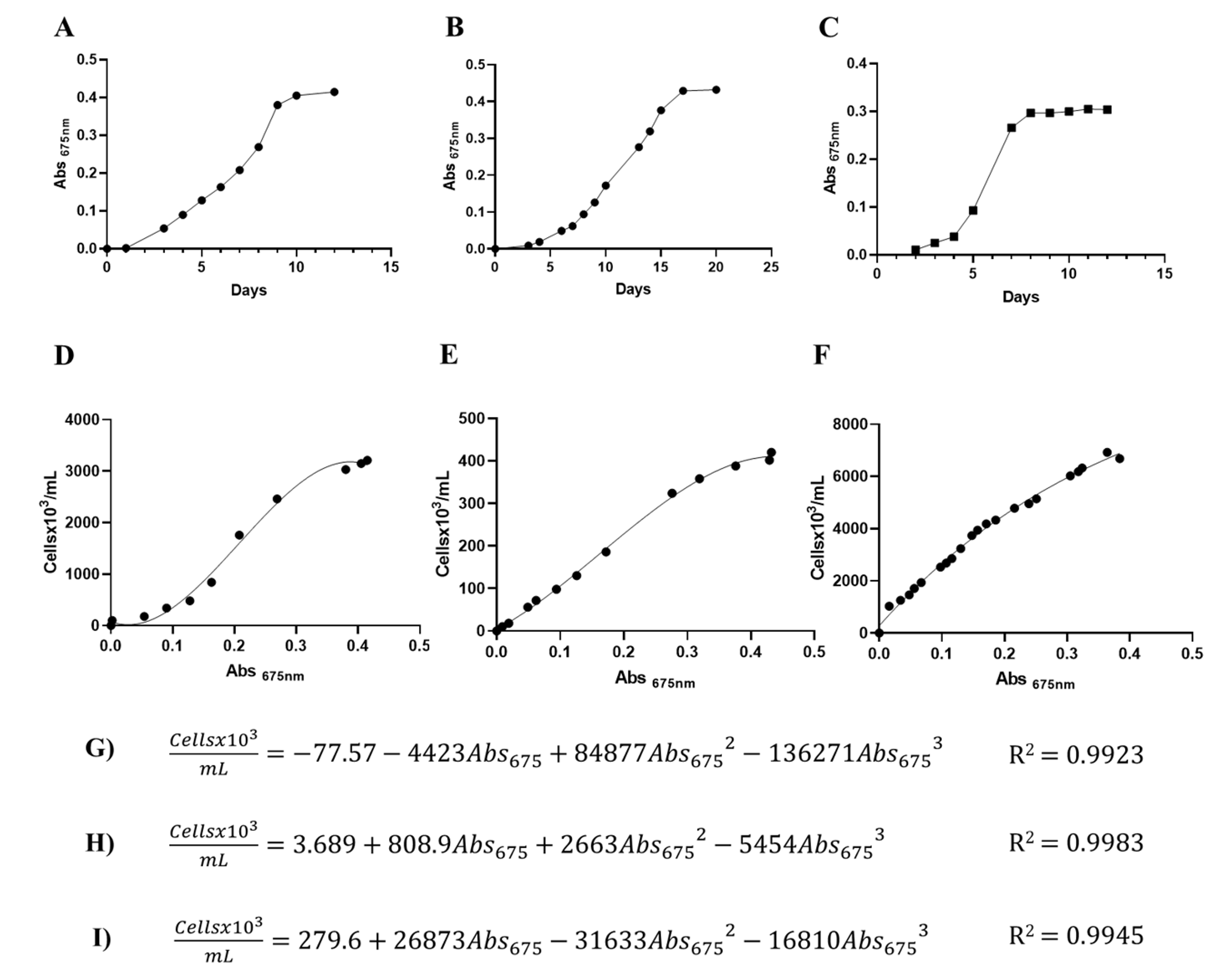

Supplement: Supplementary file 5 — Figure S3. Growth curves for 3 microalgal species in the modular photobioreactor Antares I followed by their absorption at 675 nm. Upper panels: Chlamydomonas reinhardtii in TMP medium plus air injection of 20 µL/s with 50 µmol photons m-2 sec-1 (400–700 nm) (A), Euglena gracilis in TMP medium plus 5.6 µL/s CO2 injection with 50 µmol photons m-2 sec-1 (400–700 nm) (B) and Phaeodactylum tricornutum in ESAW medium plus air injection of 40 µL/s with 50 µmol photons m-2 sec-1 (400–700 nm) (C). Middle panels: nonlinear correlation between the data of cell count growth (see Figure 2) and 675nm absorption growth curve. Chlamydomonas reinhardtii (D), Euglena gracilis (E) and Phaeodactylum tricornutum (F). Lower panels: equations of the determined third-degree order polynomial function for each species. Chlamydomonas reinhardtii (G), Euglena gracilis (H) and Phaeodactylum tricornutum (I). (PNG 449 kb) [file 12010_2023_4629_Fig9_ESM.png]

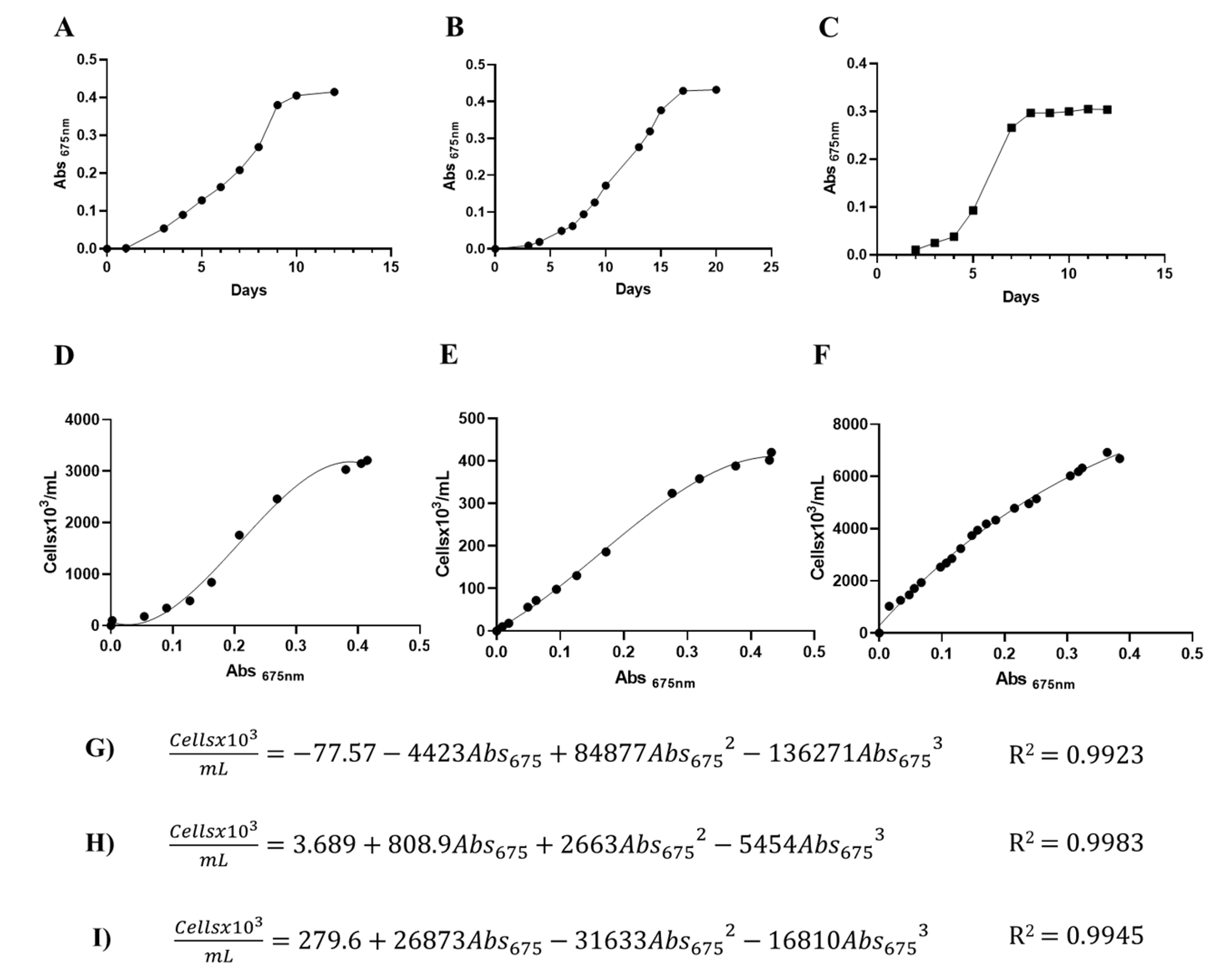

Supplement: Supplementary file 6 — High resolution image (TIF 16835 kb) [file 12010_2023_4629_MOESM3_ESM.tif]

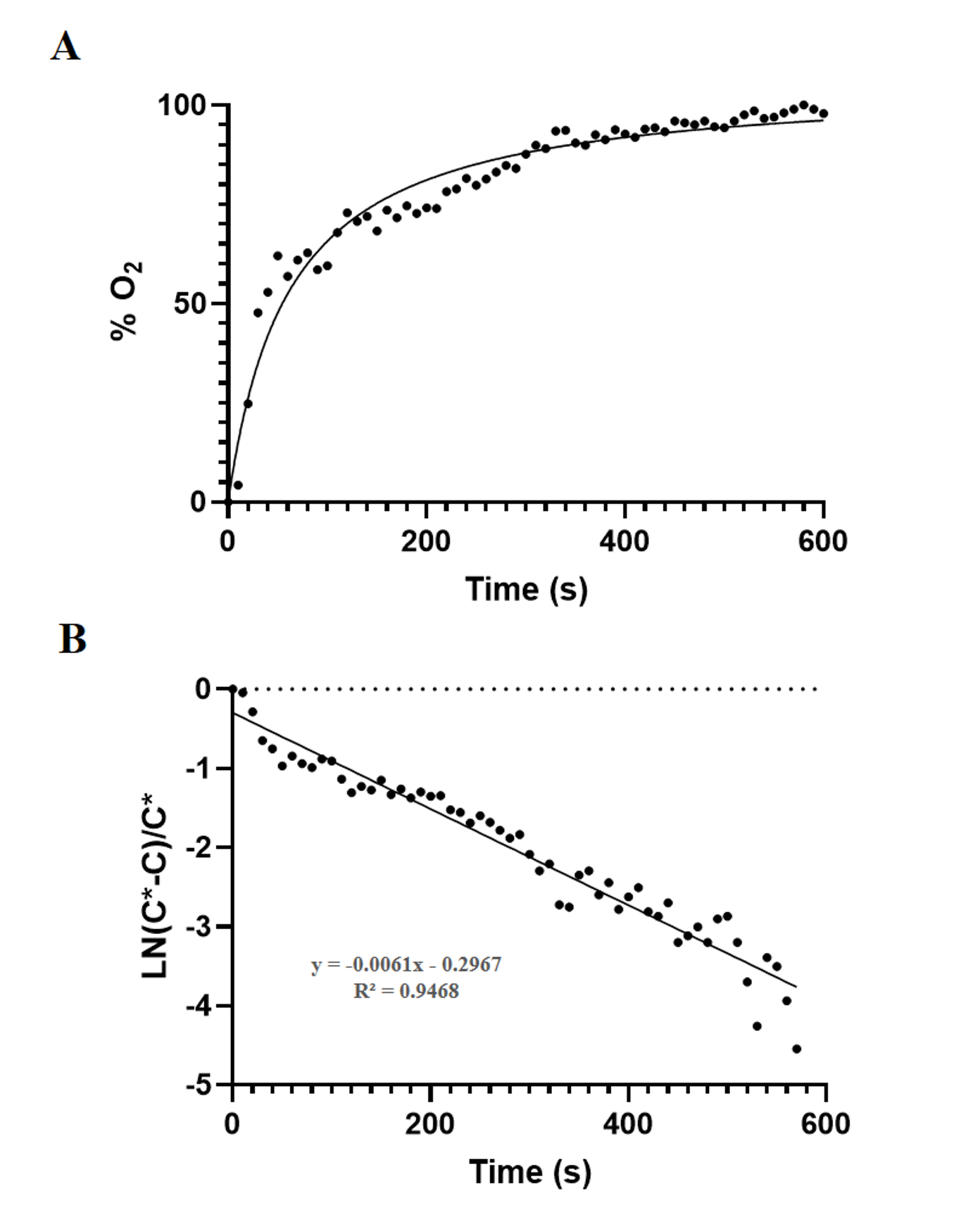

Supplement: Supplementary file 7 — Figure S4. Volumetric oxygen mass transfer coefficient (kLa) for the PBR. Dissolved oxygen quantification after the start of air injection inside oxygen-lacking media (A). Linear regression of relative oxygen concentration [(C*-C)/C*] against time. C*: Saturation oxygen concentration (8.2 mg/L); C: measured oxygen concentration by time. (PNG 187 kb) [file 12010_2023_4629_Fig10_ESM.png]

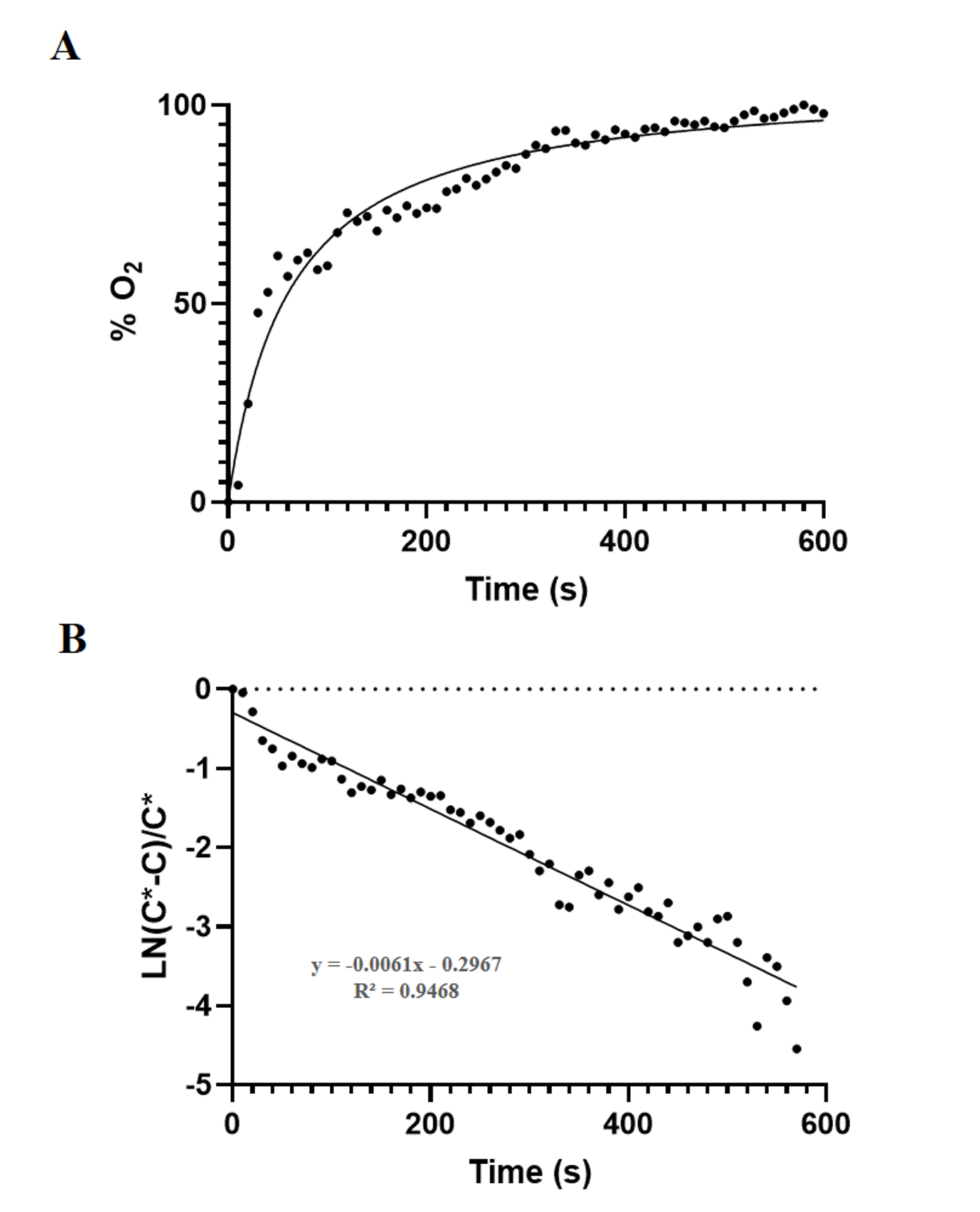

Supplement: Supplementary file 8 — High resolution image (TIF 9377 kb) [file 12010_2023_4629_MOESM4_ESM.tif]

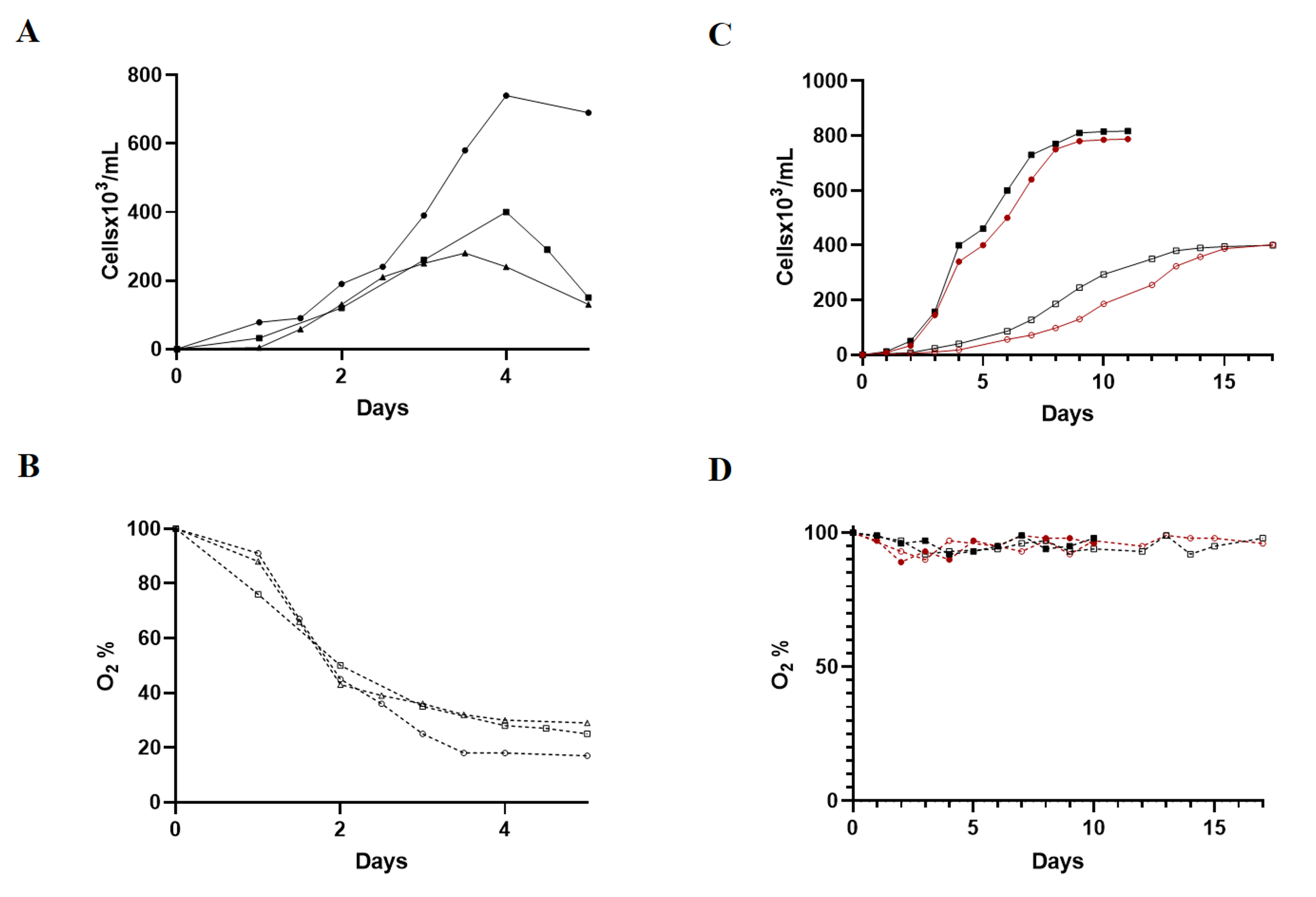

Supplement: Supplementary file 9 — Figure S5. Growth curves for 2 microalgal species in the modular photobioreactor Antares I with different gas injections. Polytomella parva in TAP medium with increasing air fluxes 25 (▲), 37 (■) and 50 (●) µL/s) (A). Dissolved oxygen concentration of the growth curves from panel A, air fluxes 25 (Δ), 37 (□) and 50 (○) µL/s) (B). Euglena gracilis in TMP medium plus 5.6 µL/s (open symbols) and 10.2 µL/s (closed symbols) of CO2 injection under different light sources, 50 µmol photons m-2 sec-1, black lines: white light, red lines: far-red light (720-730 nm) (C). Dissolved oxygen concentration of the growth curves from panel C (D).(PNG 297 kb) [file 12010_2023_4629_Fig11_ESM.png]

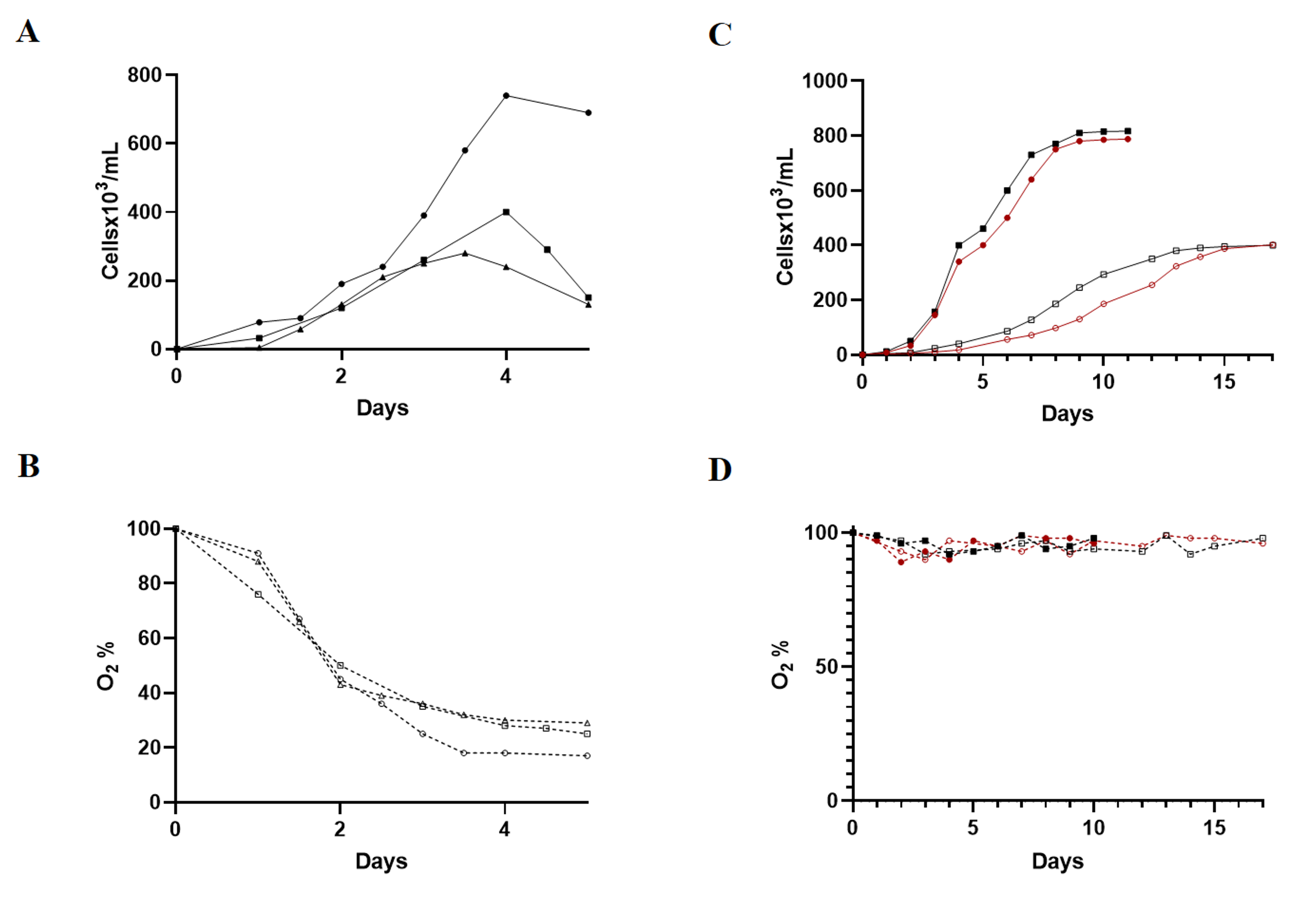

Supplement: Supplementary file 10 — High resolution image (TIF 15064 kb) [file 12010_2023_4629_MOESM5_ESM.tif]
