# Supplementary material for: Antares I: a Modular Photobioreactor Suitable for Photosynthesis and Bioenergetics Research
Source: Appl Biochem Biotechnol. 2023 Jul 24;196(4):2176–95. doi: 10.1007/s12010-023-04629-0 (PMC11035454; doi:10.1007/s12010-023-04629-0)
Supplement: Supplementary file 11 — (DOCX 14 kb) [file 12010_2023_4629_MOESM6_ESM.docx]

**Table S1.** List of items used in the design and construction of the modular PBR Antares I

| **Module** | **Item** | **Brand** | **Quantity** | **Cost (USD)** |
| --- | --- | --- | --- | --- |
| *Culture module* | Large glass vessel | *In-house* design | 1 | 400.00 |
|  | Small glass vessel | *In-house* design | 1 | 60.00 |
|  | Large glass heat interchanger U-tube | *In-house* design | 1 | 15.00 |
|  | Small glass heat interchanger U-tube | *In-house* design | 1 | 12.50 |
|  | Magnetic stirrer | Intlab | 2 | 65.00 |
|  | Scaffold | *In-house* design | 1 | 120.00 |
| *Temperature regulation module* | C-250 water chiller | Boyu | 1 | 1200.00 |
|  | SP-2500 water pump | Boyu | 1 | 26.00 |
|  | SP-1800 water pump | Boyu | 2 | 19.00 |
|  | Silicon pipeline 16 mm | Quickun | 10 | 15.30 |
| *Gas injection module* | A-807 air pump | Elite-Hagen | 2 | 37.5 |
|  | *In-situ* CO_2_ generator | ZRDR | 1 | 125.00 |
|  | 0.22 µm PVFD filter | Corning | 2 | 17.50 |
|  | GA-105 ceramic/glass CO_2_ Atomizer | Dymax | 2 | 32.5 |
|  | Bubble counter | ZRDR | 2 | 11.50 |
|  | Silicon pipeline 5mm | Boyu | 10 | 3.30 |
| *Light system module* | FLCLED 4W/25cm | Technolite | 4 | 15.00 |
|  | FLCLED 7W/45cm | Technolite | 4 | 17.50 |
|  | 12V 0.5W 5630-type color LED modules | HitLights | 80 | 0.98 |
|  | 3W LEDs far-red | Chanzon | 16 | 0.76 |
|  | APC-25-700 source | Media Well | 1 | 17.50 |
|  | APC-16-700 source | Media Well | 1 | 10.80 |
|  | 360w - 12v source | NovaLight | 1 | 50.50 |
| *Sampler module* | Peristaltic pump | Intlab | 2 | 29.55 |
|  | 3-way Luer lock | Hermed | 2 | 6.67 |
|  | Silicon pipeline 5mm | Quickun | 10 | 3.30 |
